# A review of the psychometric performance of the EQ-5D in people with urinary incontinence

**DOI:** 10.1186/1477-7525-11-20

**Published:** 2013-02-18

**Authors:** Sarah Davis, Allan Wailoo

**Affiliations:** 1 HEDS, ScHARR, The University of Sheffield, Regent Court, 30 Regent Street, Sheffield, S1 4DA, UK

**Keywords:** Urinary incontinence, EQ-5D, Quality of life, Utility, Quality adjusted life years, Psychometrics

## Abstract

Urinary incontinence can cause embarrassment and can impact on daily activities and quality of life. Generic health related quality of life instruments, such as the EQ-5D, are designed to be applicable across a variety of disease areas. However, it is sometimes claimed that they are not applicable to a certain disease area because they are missing a domain which directly captures the impact of that particular disease. For example, none of the domains of the EQ-5D relate directly to incontinence, although the impact of incontinence on quality of life may be expected to be picked up indirectly through changes in domains such as usual activities or anxiety/depression. The objective of this review was to examine the appropriateness of the EQ-5D in people with urinary incontinence by reviewing published evidence relating to the psychometric performance of the EQ-5D. A systematic search was conducted to identify studies reporting data that permitted assessment of the construct validity, responsiveness or reliability of the EQ-5D in people with urinary incontinence. Included papers were those that reported EQ-5D alongside other measures of health related quality of life or clinical measures in patients with urinary incontinence or in a broader population where results were reported for a subgroup of patients with urinary incontinence. Data were extracted and a narrative synthesis was undertaken. Seventeen papers were included in the review. In most of the tests performed, EQ-5D was consistent with clinical or disease specific outcome measures. The EQ-5D demonstrated validity in the majority of ‘known group’ comparisons, although statistical significance was not always reported. Correlations between the EQ-5D and disease specific outcomes were statistically significant and in the expected direction for most but not all of the disease specific instruments and clinical measures. For responsiveness, there was general agreement between changes in EQ-5D and changes in clinical or disease specific measures. Evidence on reliability was limited to one study. The EQ-5D was generally found to perform well on tests of construct validity, responsiveness and reliability, in people with urinary incontinence although no definitive conclusion can be made on its appropriateness based on these measures alone.

## Review

### Introduction

Urinary incontinence (UI) has been defined by the incontinence society as “the complaint of any involuntary urinary leakage” [1]. UI can cause embarrassment and can impact on daily activities and quality of life [2,3]. It can lead to depression, anxiety and can carry considerable health care costs [4]. UI is often categorised as either stress, urge or mixed. Stress incontinence is associated with effort, exertion, sneezing or coughing, whilst urge incontinence is when leakage is accompanied or immediately preceded by urgency. The term mixed incontinence is used when features of both stress and urge incontinence are present.

Treatments which improve continence may have a beneficial impact on the individual’s health related quality of life (HRQoL). Reimbursement agencies are interested in knowing the impact of treatment on HRQoL when making decisions regarding whether a treatment should be made available within their health care system. Often these decisions are informed by cost-utility analyses in which treatment benefits are expressed as a change in quality adjusted life years (QALYs). QALYs are useful as they facilitate comparisons of health benefits across different interventions, patients and disease areas. In order to calculate treatment benefit in terms of QALY gains, an estimate of health utility is required. Health utility is a single metric for HRQoL, where one represents a state of full health and zero represents a state equivalent to death. Negative values are possible as these represent states that are considered to be worse than death. Whilst there are a variety of generic and disease specific instruments available to measure HRQoL, only a few of these provide the preference based measurement of health utility required for cost-utility analyses.

One of the most widely used generic preference based instruments is the EQ-5D. The EQ-5D is a generic instrument intended to measure and value health outcomes across a wide range of diseases and treatments. It is therefore described as a generic rather than a condition specific instrument. It consists of two main components. First, a classification or descriptive system that covers five health domains: mobility, self-care, usual activities, pain/discomfort and anxiety/depression. The standard and most widespread version of the EQ-5D has three levels: no problems, some problems, severe problems. There are therefore 243 health states that can be described in what is generally accepted as a simple approach to describing health. Second, a single valuation (EQ-5D index or tariff) is provided for each particular health state in the descriptive system. The EQ-5D is the preferred instrument for measuring health utilities in adults within the Technology Appraisals Programme at the National Institute for Health and Clinical Excellence (NICE) [5].

Whilst generic HRQoL instruments are designed to be applicable across a variety of disease areas, it is sometimes claimed that they are not applicable to a certain disease area because they are missing a domain which directly captures the impact of that particular disease. In the case of UI, the EQ-5D lacks any domain that directly relates to continence, although the impact of incontinence on HRQoL may be expected to be picked up indirectly through changes in domains such as usual activities or anxiety/depression. Evidence is therefore needed on the appropriateness of the EQ-5D in this setting. Psychometric methods are often employed to inform assessment of the appropriateness of an instrument for use within a particular population. The aim of this review was to examine the appropriateness of the EQ-5D for measuring health utility in people with UI by examining all published evidence relating to the psychometric performance of the EQ-5D.

### Methods

#### Search strategy and data extraction

The search strategy combined free text terms aimed at identifying papers reporting EQ-5D with free text and controlled terms (MESH and MESH-like terms) for UI. The following databases were searched in May 2010; BIOSIS, CINAHL, Cochrane Library (comprising CDSR, CENTRAL, NHS EED), EMBASE, Euroqol website, MEDLINE, PsychNFO, Web of Science. The search strategy for MEDLINE is provided in the Additional file 1.

Included papers were those that reported EQ-5D alongside other measures of HRQoL or clinical measures in patients with UI or in a broader population where results were reported for a subgroup of patients with UI. Papers reporting valuations of clinical vignettes were excluded. There were no restrictions relating to study design or interventions. Relevant systematic reviews and economic evaluations were ordered and their references checked for additional papers reporting primary data. Only English language studies were reviewed. Titles and abstracts were sifted by two reviewers independently with discussion used to resolve any inclusion / exclusion discrepancies. Full text papers were sifted by a sole reviewer.

Data were extracted using a standardised set of forms. Data extracted included study characteristics (country, study design, type of incontinence and severity measures, treatment where relevant), participant characteristics (number, age, gender, ethnicity), outcome measures and results of psychometric tests.

#### Psychometric measures

When establishing the appropriateness of a HRQoL instrument within a particular disease area, relevant psychometric properties include acceptability, feasibility, reliability, validity, and responsiveness [6]. The concept of validity refers to the extent to which an instrument measures what it is intended to measure, but in this case, all measures of validity are limited by the fact that there is no gold standard measure of health utility against which to judge performance. Brazier and Deverill (1999) identify several criteria that psychometricians use to measure validity in the absence of a gold standard measure [6]. ‘Known group validity’ examines differences between groups which are known to differ in the concept of interest, e.g health utility. Given the lack of a gold-standard measure of health utility, in practice the groups are often defined in terms of clinical measures such as disease severity. ‘Convergent validity’ refers to the situation where an instrument is highly correlated with other instruments which measure the same underlying construct. ‘Discriminant validity’, is where measures that theoretically should not be related to each other are observed not to be correlated with each other. Known-group, convergent and discriminant validity are all measures of construct validity. Other forms of validity such as face validity and content validity are concerned with whether the items of the instrument are appropriate for the health dimension being measured, in this case the conceptual model of health that is accepted to define the “quality of life” element of QALY calculations. These measures would need to be assessed in a broader population than considered here. Responsiveness refers to the ability of an instrument to reflect changes that occur in patients over time and therefore requires the comparison of longitudinal data in groups that are known to have changed in the concept of interest. Reliability can be thought of as the stability of results when using an instrument repeatedly in situations where the results are not expected to change, such as over time in the same unchanged population (test-retest reliability), or between raters or interviewers (inter-rater reliability). The acceptability and feasibility of the EQ-5D is well established and is not expected to be significantly different for this population, so the review was limited to measures of construct validity, reliability and responsiveness.

### Results

**Figure 1:**
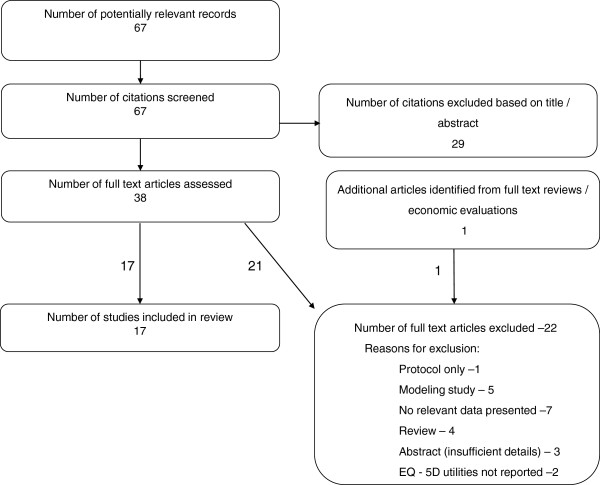
Identification of included articles.

A total of 67 citations were identified from the bibliographic searches (Figure 1). Of these 38 were ordered as full-text articles, although nine papers (four reviews and five economic evaluations) were ordered purely to check their references for further primary studies. From these one further paper was identified.

A total of 17 papers were included in the review, the key features of which are reported in Table 1. Four of the studies identified were randomised controlled trials (RCTs), four were cohort studies and nine were cross-sectional studies. None of the studies were specifically designed to assess the psychometric properties of the EQ-5D. One paper reported that its objective was to evaluate the measurement properties of the EQ-5D using data collected as part of a RCT [7]. Two further studies aimed to validate another HRQoL instrument [2,8].

**Table 1:** Characteristics of included studies

**Author(s), Year**	**Country**	**Type of incontinence (e.g stress, urge)**	**Treatment (if any)**	**Study type (e.g. cross sectional, RCT, cohort)**	**Number of participants**
Ternent et al, 2009 [20]	UK	Stress incontinence	No details	Cross sectional (self-selected sample)	105 (of 188 approached)
Ismail et al, 2009 [16]	UK	Urodynamic stress incontinence	Magnetic energy stimulation of pelvic floor muscles	Cohort	48
Rinne et al, 2008 [22]	Finland	Stress UI with indications for surgical treatment	a) Tension-free vaginal tape (TVT)	RCT	267 (of 273 randomised)
			b) TVT obturator (TVT-O)		
Haywood et al, 2008 [7]	UK	Stress and/or urge incontinence in women referred for physiotherapy from primary or secondary care.	Physiotherapy	Cohort (RCT with data combined across arms)	174
Monz et al, 2007 [12]	15 European Countries (UK and Ireland subgroup)	UI of any type in women seeking treatment	At discretion of physician	Cross-sectional data from cohort study	9487
Kobelt et al, 2006 [21]	France, Germany, Italy, Sweden, UK	Stress UI	NASHA/Dx gel	Cohort	82 of 139 enrolled
Dumville et al, 2006 [17]	UK	Proven stress UI requiring surgery	Laparoscopic vs open colposuspension	RCT	291
Currie et al , 2006 [10]	UK	Stress and non-stress incontinence in patients identified from sample which had been treated by urology department.	None specified	Cross-sectional	609 (from 2193 sent survey)
Monz et al, 2005 [13]	15 European countries	UI in women seeking treatment	None	Cross-sectional data from a cohort study	9487
Manca et al, 2003 [18] (clinical outcomes from Ward 2002)	UK	Stress incontinence with indication for surgical management	Tension-free vaginal tape vs colposuspension	RCT	344
Kobelt, 1997 [14]	Sweden	Mixed or urge incontinence in patients who had previously received therapy from a urotherapist.	None specified	Cross-sectional	461 (541 sent questionnaire)
Hawthorne, 2009 [2]	Australia	General population sample with data on presence and severity of UI	None	Cross-sectional	3015
Tincello et al, 2010 [19]	Germany, UK, Sweden & Ireland	Stress UI, with or without urge symptoms, in women seeking treatment	36.1% receiving conservative management at baseline. 18.0% receiving drug therapy at baseline.	Cross-sectional (baseline data from cohort study)	3739 of 3762 enrolled
Saarni, 2006 [9]	Finland	Self-reported UI in general population sample	None	Cross-sectional	8028 of which 13.0% reported UI
Noble et al, 2002 [11]	UK	Uncomplicated urinary tract symptoms in men with benign prostatic enlargement	Laser therapy vs Transurethral prostrate resection vs conservative management	RCT	340

Mihaylova et al, 2010 [23]	Multicountry	Stress UI	Duloxetine vs conservative management vs duloxetine plus conservative management vs no treatment	Cohort (non randomised comparison of treatments)	1510
	(Germany, UK & Sweden)	40% had pure stress incontinence with the rest reporting both stress and urge incontinence			
Donovan et al, 1997 [8]	12 countries	Outpatients attending urology department with symptoms (not specifically incontinence) and possible benign prostatic obstruction. GP sample (not selected for condition)	None	Cross-sectional	1271 outpatient sample
					423 GP sample (UK)

GP=General Practice NASHA/Dx =non-animal-stabilized hyaluronic acid/dextranome, RCT=randomised controlled trial, UI=urinary incontinence, UK=United Kingdom.

The majority of the studies were conducted in a population with incontinence. In two studies, a sample of the general population were asked whether they had a range of clinical conditions including incontinence [2,9]. These studies were included as they reported utilities for the subgroup of patients with incontinence. One study identified patients from an academic urology unit inpatient database and examined overactive bladder symptoms including incontinence [10]. One study was in men with uncomplicated urinary tract symptoms associated with benign prostatic enlargement [11]. A second study was conducted in outpatients attending a urology department with urinary symptoms (not specifically incontinence) and possible benign prostatic obstruction [8]. This study also recruited a general practice sample which was not selected for incontinence [8]. These studies were included as UI can be experienced in patients with benign prostatic hyperplasia. Two papers reported different analyses from the Prospective Urinary Incontinence Research (PURE) study [12,13]. One paper reporting EQ-5D values from a study [14] had a second associated paper [15] which was excluded as it didn’t report EQ-5D values, however the EQ-VAS values reported in this secondary paper are included in the results table under the primary paper.

One study enrolled less than 100 patients [16]. The total number of patients ranged from 48 to 9487. The mean age across the cohorts with UI varied from 50 to 67. One study reported a higher mean age in the patients reporting UI than in the general population sample as a whole (mean age of 64 versus 53) [9], whilst another reported only the mean age for the general population sample [2]. Two papers looked exclusively at males [8,11], four had a mixed population of males and females [2,9,10,14], and the remainder looked exclusively at females. Ethnicity was reported in a single study in which 4% of participants were non-white [10].

The measures reported in each of the included studies are shown in Table 2 (all abbreviations used to describe HRQoL instruments are defined below Table 2). In addition to the EQ-5D, five studies administered the SF36 or some variant of it [8,10,14,17,18]. One included SF-6D, AQoL, AQoL-8, and HUI-3 [2] and one reported the 15-D [9]. Several papers reported using the UK valuation set for the EQ-5D and none reported using an alternative valuation set, although it was common for this information not to be reported. Only two studies reported the EQ-VAS [12,14].

**Table 2:** Measures reported in the included studies

	**Generic measures**		**Other measures used**			
**Author(s), Year**	**Descriptive system**	**Tariff used**	**Direct valuation**	**Condition-specific HRQoL measures used**	**Clinical measures used**	**Qualitative questions**
Ternent et al, 2009 [20]	EQ-5D	Not stated	None	KHQ	None	None
				PGI		
Ismail et al, 2009 [16]	EQ-5D	Not stated	None	KHQ	1 hr pad test	None
					Leakage episodes	
					Pad usage	
Rinne et al, 2008 [22]	EQ-5D	Not stated	None	UISS	Cough stress test	Satisfaction with operation.
				DIS	24-hr pad	
				VAS		
				IIQ-7		
				UDI-6		
Haywood et al, 2008 [7]	EQ-5D	States general population utility weights.	None	I-QoL (index and individual domains)	SSI	Subjective treatment benefit assessed by patient.
					Incontinence episodes per week at baseline	
Monz et al, 2007 [12]	EQ-5D	Not stated	EQ-VAS	I-QOL	UI severity (Sandvik Index)	Bother (4 point scale)
					UI subtype (S/UIQ)	
Kobelt et al, 2006 [21]	EQ-5D	Reference suggests UK tariff used.	None	None	Incontinence grade	
					Median number of episodes per day	
Dumville et al, 2006 [17]	EQ-5D	UK tariff	None	None	Objective cure* (negative 1 hr pad test)	Subjective cure* (perfectly happy / pleased) to spend rest of life with current urinary symptoms
	SF-36					
					*(reported in related clinical paper)	
Currie et al, 2006 [10]	EQ-5D	Not stated	None	None	None	None
	SF-36					
Monz et al, 2005 [13]	EQ-5D	Not stated	None	I-QOL	Sandvik index (severity based on frequency and leakage amount)	Bothersomeness and limitations of daily activities
Manca et al, 2003 [18]	EQ-5D	UK tariff			Objective cure (based on negative pad test and negative cystometry)	
	SF-36					
					Subjective cure (based on BFLUTS)	
Kobelt, 1997 [14]	EQ-5D	UK tariff.	EQ-VAS [15]		Frequency of micturitions and involuntary urine loss (combined measure)	
	SF-36					
Hawthorn, 2009 [2]	EQ-5D	EQ-5D: UK tariff				
	SF-6D					
	AQoL	SF-6D: Not stated				
	AQoL-8 (derived from					
		AQoL &				
	AQoL)	AQoL-8: community TTO				
	HUI-3 (deciles)					
Tincello et al, 2010 [19]	EQ-5D	UK tariff	None	None	Episodes per week	None
Saarni, 2006 [9]	EQ-5D	EQ-5D: UK tariff		None	None	None
	15-D					
		15-D Finnish valuation set				
Noble et al, 2002 [11]	EQ-5D	Not stated	None	I-PSS which includes a quality of life score.	Maximum flow rate	
					Post void residual urine	
					Number of successful procedures (based on I-PSS and maximum urinary flow)	
Mihaylova et al, 2010 [23]	EQ-5D	UK tariff			Number of leaks during 7 days	
Donovan et al, 1997 [8]	EQ-5D (UK, Denmark and Netherland only, N=359)	Not reported		ICSQol (ICSmale)		
	SF-36 (UK only, N=205)					

AQoL=Assessment of Quality of Life, BFLUTS=Bristol Female Lower Urinary Tract Symptoms Questionnaire, DIS= Detrusor instability scores, EQ-VAS=Visual analogue scale which accompanies the EQ-5D descriptive system, HUI-3=Health Utilities Index Mark 3, ICSQol=International Continence Society – Benign Prostatic Hyperplasia study Quality of Life Instrument, IIQ-7=Incontinence Impact Questionnaire-short form, I-PSS = International Prostate Symptom Score, I-QOL=Incontinence specific Quality of life Questionnaire, KHQ=King’s Health Questionnaire, PGI = Patient Generated Index, SF-36=Medical outcomes study 36-Item Short-Form Health Survey , SF-6D= Classification for describing health derived from a selection of SF-36 items, SSI=Symptom Severity Index, S/UIQ=Stress and Urge Incontinence Questionnaire, UDI-6=Urogenital Distress Inventory-short form, UI=Urinary incontinence, UISS=Urinary Incontinence Severity Score, VAS=Visual Analogue Scale, 15-D=Fifteen dimension generic instrument.

The main clinical measures reported were severity, or grade of incontinence, type of incontinence (stress / urge / mixed), frequency of leakage episodes and pad usage or pad tests to determine volume of leakage. Some studies reported on cough stress tests or cystometry results. In the benign prostatic hyperplasia populations maximum flow rate and post void residual volume were used as measures of treatment effectiveness.

Various symptom scoring and incontinence specific quality of life tools were also used (KHQ, UISS, I-QOL, IIQ-7, SSI). Some studies included tools which were designed for use in patients with overactive bladder rather than incontinence (UDI-6, BFLUTS). Some studies included scales designed to measure the impact of lower urinary tract symptoms in men (ICSQoL, IPSS). One study reported a questionnaire that assesses the likelihood of destrusor instability (DIS) which may be associated with stress incontinence, based on patient history. One study reported quality of life using a patient generated index (PGI) which is an individualised health related quality of life measure.

#### ‘Known group’ validity

A summary of those studies that compared the mean EQ-5D between groups defined in terms of incontinence severity, frequency or type of incontinence is provided in Table 3.

**Table 3:** Results of ‘known group’ comparisons

**Author(s), Year**	***Groups defined as***	***Instrument***	***Direction of change consistent across groups and consistent with clinical expectation?***	***Difference between groups statistically significant?***
Haywood et al, 2008 [7]	Number of episodes at baseline:			
		EQ-5D	Yes‡	No at p=0.01
	Not at all	SSI	Yes	Yes, p<0.01
	A few days	I-QoL index	Yes	Yes, p<0.01
	Half the week	I-QoL domains	Mixed†	Yes, p<0.01
	Most days			
	Every day			
Tincello et al, 2010 [19]	Episode frequency:			
	<=7 per week	EQ-5D	Yes	Yes, p<0.0001
	7 to 13 per week			
	>=14 per week			
Monz et al, 2005 [13]	Severity (reported for each subtype)			
	Slight	EQ-5D	Yes	Not reported
	Moderate	EQ-VAS	Yes	Not reported
	Severe	Mean I-QoL	Yes	Not reported
	Very severe	I-QoL domains	Yes	Not reported
Hawthorne, 2009 [2]	Continence status:			
	a) None	EQ-5D	Yes	Yes, p<0.0001
	b) Slight/mild	SF-6D	Yes	Yes, p<0.0001
	c) Moderate	AQoL	Yes	Yes, p<0.0001
	d) Severe	AQoL-8	Yes	Yes, p<0.0001
Currie et al, 2006 [10]	Type of incontinence:			
	General	EQ-5D	Stress<general<none*	Not reported
	Stress	SF-36	As for EQ-5D	As for EQ-5D
	None			
Monz et al, 2005 [13]	Subtype (reported for each severity category):			
		EQ-5D	Stress>urge>mixed*	Not reported
		EQ-VAS	As for EQ-5D (except when severity slight)	Not reported
	Stress			
		Mean I-QoL	As for EQ-5D	Not reported
	Urge	I-QoL domains	No consistent pattern across all domains	Not reported
	Mixed			
Tincello et al, 2010 [19]	UI subtype:			
	Mixed	EQ-5D	Stress>urge>mixed*	Yes, p<0.0001
	Pure stress			
	Pure urge			

†Yes for 2/3 domains, ‡Same mean for two least severe domains, *Unclear which type of incontinence is expected to have lower utility. VAS=visual analogue scale, I-QOL=Incontinence specific Quality of life Questionnaire, SSI= symptom severity index, SF-36=Medical outcomes study 36-Item Short-Form Health Survey, SF-6D= Classification for describing health derived from a selection of SF-36 items, AQoL= Assessment of Quality of Life.

Two studies defined groups by the frequency of incontinence episodes [7,19]. In one study, three groups were defined and the mean EQ-5D consistently reflected differences between groups and the differences were statistically significant [19]. In the second study, five groups were defined [7]. The mean EQ-5D was equal for two of the groups and the differences between all the five groups were not statistically significant. In the same study, the condition specific measures of SSI and I-QoL discriminated well between the groups.

Two studies reported ‘known group’ validity by severity group. In one study the definition of severity was not well described [2], but in the other [13] a validated severity index was used which was based on combined scores for frequency and leakage amount. EQ-5D varied between severity groups as expected in both studies and had statistically significant differences between severity groups in one study [2], whilst the other did not report whether differences were statistically significant [13]. Other preference based measures (SF-6D, AQoL & AQoL-8), generic measures (EQ-VAS) and disease specific measures (I-QoL) were found to perform equally well.

Three studies compared groups defined by incontinence type with two studies distinguishing between stress, urge and mixed incontinence [13,19] and the other study grouping patients as general incontinence, stress incontinence or none [10]. It was unclear what differences were clinically expected between the stress, urge and mixed groups. However, two studies reported greater EQ-5D scores for stress incontinence than for urge and greater utilities for urge than for mixed [13,19]. These differences were statistically significant in one study and the other did not report statistical significance. EQ-VAS had differences across the groups that were consistent with the differences for EQ-5D except for when severity was reported as slight. Mean I-QoL score performed similarly to EQ-5D although the differences between the groups were not consistent for individual I-QoL domains.

In the third study EQ-5D scores were lower for general incontinence than for no incontinence as clinically expected, but statistical significance was not reported [10]. SF-36 performed equally well in distinguishing between UI type which was categorised as general / stress / none.

#### Convergent validity

Five studies provided information on the correlation between EQ-5D and disease specific instruments (KHQ, PGI, I-QoL, ICS-QoL, SSI) or clinical measures (incontinence grade and number of micturitions / leakages). Significant correlations in the expected direction were seen for several but not all of the disease specific instruments. One study reported a statistically significant correlation (p<0.01) in the expected direction for both the I-QoL index and the three I-QoL scale scores [7]. In the same study, SSI was found not to have a statistically significant correlation with EQ-5D (p>0.05) [7]. The correlations between EQ-5D and the individual ICS-QoL items were all in the expected direction but were not all statistically significant [8]. One study reported significant correlations in the expected direction for PGI and KHQ, but p-values were not specified [20]. Significant correlations were found with incontinence grade (p<0.05) [21] and the number of micturitions and leakages (p<0.001) [14].

Two studies used regression techniques to assess the impact of clinical measures on EQ-5D scores. Severity, subtype of incontinence (e.g stress / urge) and number of episodes were found to be significant predictors [12,19]. Two studies used multivariate regression to examine whether presence of incontinence was a significant predictor of utility. The first found that presence of incontinence was a significant predictor of EQ-5D in urology patients and was also a significant predictor of SF-36 scores [10]. The second study found that incontinence was a significant predictor of both EQ-5D and 15D in a general population sample and the size of utility loss was similar between these two instruments [9].

#### Responsiveness

Results from studies that provide details on the responsiveness of EQ-5D in incontinence are reported in Table 4. Five studies reported changes in EQ-5D from baseline and compared this to changes in disease specific or clinical measures [11,16,18,21,22]. Generally there was agreement between changes in EQ-5D and changes in clinical or disease specific measures with four studies reporting improvements in both [11,18,21,22] although two studies did not report whether the EQ-5D changes were statistically significant [11,18]. In one study there was no significant change in either EQ-5D or clinical outcomes [16].

**Table 4:** EQ-5D responsiveness results

**Author(s), Year**	***Comparison***	***Change in clinical measure(s) or other preference based utility***	***Change in EQ-5D***	***Agreement with direction?***	***Agreement with statistical significance?***
Ismail et al, 2009 [16]	Change over time	No significant change on any measure (KHQ,1 hr pad test, pad use, leakage episodes)	No significant change	NA	Yes
Rinnie et al, 2008 [22]	Change over time	24 hr pad test significantly improved in both arms	Significant improvement in both arms	Yes	Yes
		All condition specific measures (UISS, DIS, VAS, IIQ-7, UDI-6) significantly improved in both treatment groups			
		EQ-VAS significantly improved in both treatment groups			
	Difference between treatment arms	No significant difference in objective cure, leakage, complication rate, UISS, DIS, VAS, IIQ-7, UDI-6.	No significant difference in EQ-5D	Agreement with some clinical outcomes and not others.	Yes
Haywood et al 2008 [7]	Comparison of means for responders and non-responders	6 week data:	6 week data:	6 week data:	6 week data:
		SSI and I-QoL index had difference in expected direction but not statistically significant (at p=0.01). Two of the I-QoL domains had significant difference.	EQ-5D had difference in expected direction but not statistically significant (at p=0.01).	Yes	Not consistent with all
		
		
		5 mth data:	5 mth data:	5 mth data:	5 mth data:
		As for 6 weeks except only one of the I-QoL domains had significant (p<0.01) difference.	EQ-5D had difference in expected direction and statistically significant (p=0.01).	Yes	Not consistent with all.
		
	Mean change scores for patients reporting improvement	6 week data:	6 week data:	6 week data:	6 week data:
		Expected direction and significant (at p=0.05) for SSI, I-QoL index, I-QoL domains	Expected direction but p>0.05	Yes	No
		5 mth data:	5 mth data:	5 mth data:	5 mth data:
		As for 6 weeks but larger changes.	Expected direction and p<0.05.	Yes	Yes
	MSRM for patients reporting improvement	6 week data:	6 week data:	6 week data:	6 week data:
		SSI, 0.70	0.07	Yes	No
		I-QoL index, 1.01			
		I-Qol domains, 0.40 to 0.94			
		5 mth data:	5 mth data:	5 mth data:	5 mth data:
		SSI, 0.67	0.26	Yes	Yes
		I-QoL index, 1.17			
		I-Qol domains, 0.80 to 1.25			
Kobelt et al, 2006 [21]	Median incontinence episodes per day for clinical outcome but change from baseline for EQ-5D	All patients:	All patients:	All patients	All patients
		3.0 at baseline, 0.7 at 3mths and 0.9 at 12 mths (p<0.0001 and p<0.001 for differences)	3 mths: 0.048 (p<0.001)6 mths: 0.014 (not significant)	3 mths: Yes	3 mths: Yes
		
			12 mths: “gain remained evident”	12 mths: Yes	12 mths: Yes
		
			Patients with utility<1 at baseline:	Patients with utility <1 at baseline:	Patients with utility <1 at baseline:
			3 mths: 0.099 (p<0.01)		
			6 mths: 0.065 (p<0.001)		
			12 mths: “significant improvements”	As for all patients	As for all patients
Dumville et al, 2006 [17]	Difference between treatment arms:	Objective and subjective cure rates and SF-36 scores showed no significant difference	QALY gain based on EQ-5D utility scores showed no significant difference (CrI crossed zero)	No change in either clinical, generic HRQoL or utility	Yes
Manca et al, 2003 [18]	Differences from baseline to 6mths	Pad weight decreased significantly for both groups.	Utility increased in both arms (significance not reported)	Yes	Not reported
		Significant reduction in leakage episodes in both groups (P<0.0001)			
		Significant reduction in 21/30 symptoms (BFLUTS) in both groups (P<0.0001)			
	Differences between trial arms:	No significant difference in objective or subjective cure rate between trial arms	QALY difference between arms based on EQ-5D scores non significant at p=0.05	Agreement with clinical outcomes but didn’t detect differences between arms in some SF-36 domains	Yes for clinical outcomes, no for some SF-36 domains
		SF-36 scores had significantly smaller improvement/ greater decline lower for colposuspension group vs TVT in four domains at 6 weeks and four domains (three same and one different) at 6 mths.			
Noble et al, 2002 [11]	Change from baseline:	Improvements in I-PSS, maximum urine flow, and residual volume were significant (p=0.05) for laser and resection but not conservative.	Means increased for laser and resection but not conservative (p values not reported)	Yes	Not reported
		Improvements in I-PSS QoL were significant for all three interventions.			
	Differences between trial arms:	Resection vs conservative and laser vs conservative showed significant difference in all four outcomes.	Gains were greater for resection than laser therapy (p values not reported)	Yes	Not reported
		Laser vs resection showed significant difference in only one outcome which was in favour of resection (maximum flow)			
Mihaylova et al, 2010 [23]	Comparison between active treatment arms and no treatment:	Number of leaks avoided per week was significantly (p<0.01) better for Duloxetine alone, conservative alone and duloxetine plus conservative (all relative to no treatment).	QALY gains based on EQ-5D utility were significant for Duloxetine alone (p<0.01) and duloxetine plus conservative treatment (p<0.05) but conservative alone was not significant and was negative (all compared to no treatment)	Yes for two of three comparisons against no treatment	Yes for two of three comparisons against no treatment
	Comparison between the three active treatment arms:	No significant reduction in number of leaks for 3 comparisons between active treatment arms.	Significant (p<0.05) QALY gains for 2 of 3 comparisons between active treatment arms.	Yes for 2 of 3 comparisons between active treatment arms.	No for 2 of 3 comparisons between active treatment arms.

MSRM=modified standardised response mean.

One study reported changes from baseline for patients whose continence-specific health improved [7]. In this subgroup significant changes from baseline were seen in SSI and I-QoL, but not EQ-5D at six weeks. However, by five months when greater changes from baseline were seen for SSI and I-QoL, the EQ-5D changes were also found to be larger and statistically significant. This study also reported mean scores for responders and non-responders with response being based on patient perceived benefit. There were significant differences between responders and non-responders in two of the I-QoL domains at six weeks, but differences in SSI, I-QoL index and EQ-5D were non-significant. However, by five months EQ-5D differences were found to be significant although only one I-QoL domain remained significantly different between responders and non-responders.

Five studies reported whether the difference between treatment groups was significant for both EQ-5D and for other measures (clinical, disease specific measures and generic HRQoL) [11,17,18,22,23]. In three studies there were no statistically significant differences in EQ-5D between treatment groups and this agreed with the other trial outcomes [17,18,22]. In one of these studies some significant differences were found in some domains of the SF-36 but not in the other clinical outcomes (objective and subjective cure rates) [18]. One study found differences in EQ-5D scores between the treatment arms that were consistent with the clinical outcomes, but the statistical significance of the EQ-5D differences was not reported [11]. In another study six comparisons were made between the four treatment options (three active and one no treatment) [23]. For the three comparisons of active treatment against no treatment, all three active treatments were more clinically effective than no treatment but only two had significantly better EQ-5D scores. For the three comparisons between the active treatment arms, no significant differences were seen in the clinical effectiveness, but there were significant differences in the EQ-5D scores for two comparisons.

One study reported standardised response means for different instruments [7]. The standardised response means were lower for EQ-5D than for disease specific measures (SSI and I-QoL).

#### Key findings on re-test reliability

One study reported the intraclass correlation coefficient (ICC) for patients reporting no benefits from treatment during a clinical trial (data from both trial arms were combined) [7]. The test-retest correlation for EQ-5D was 0.83 (n=50).

### Discussion

The EQ-5D appears to be a reasonable instrument to use in this population when considering the psychometric measures of construct validity, responsiveness and reliability. In most situations EQ-5D performs well when assessed by ‘known group’ validity or responsiveness. In most of the responsiveness tests performed, EQ-5D was consistent with clinical or disease specific outcome measures, including in achieving statistical significance. However, there were situations where statistical significance was not achieved.

Psychometric measures such as validity, reliability and responsiveness are often used to support claims that a HRQoL instrument is adequate or inadequate in a particular population. These measures rely on making comparisons between the scores achieved by the HRQoL instrument and other instruments or clinical measures which are expected to be related. However, when the instrument in question intends to measure health utility, as EQ-5D does, these comparisons are not tests. They can highlight differences between EQ-5D and other instruments such as other generic instruments, disease specific outcomes or clinical measures, but since there is no gold standard it cannot be established conclusively which measure is “right”. Intuition and judgement are required to draw any stronger conclusions. Another issue for consideration when interpreting the results is that the populations of the included studies are somewhat diverse with some studies recruiting patients specifically with symptoms of UI and other studies recruiting patients with conditions which may be associated with UI such as overactive bladder and benign prostatic enlargement.

Limitations to the studies included in the review can only further dilute the conclusions that may be drawn. In particular, none of the studies reported here were specifically designed to test the appropriateness of the EQ-5D, they simply provided data which was potentially relevant. Where studies are not explicitly powered to detect a difference in EQ-5D scores, a lack a statistical significance in a particular comparison may be related to the size of the sample rather than a reflection on the appropriateness of the EQ-5D. Further more, sometimes not all of the data relevant to assessing a particular psychometric property were provided. For example, three of the studies providing data on responsiveness were RCTs reporting changes from baseline for the EQ-5D and other clinical measures, but two did not report whether the EQ-5D changes were statistically significant.

Where known groups are defined in terms of some clinical measure, the distinctions between groups may reasonably not translate to differences in health utilities. For example, Haywood *et al*. found that EQ-5D was not able to fully discriminate between 5 groups [7]. The groups were defined in terms of the number of episodes as “not at all”, “a few days”, “half the week”, “most days” and “every day”. The differences between the groups are therefore relatively small, not necessarily mutually exclusive, and it is questionable whether there would be significant differences in the preferences of patients in some of the groups.

Furthermore, the reporting of the extent to which an instrument is consistent with groups defined in another way needs to consider how many groups are being considered. Often there are multiple groups being compared and the instrument may provide consistent results across many of them. P-values typically relate to the null hypothesis that the mean value is equal in all the subgroups under consideration. This itself may be ambiguous because it does not consider how many of the individual pairs of comparisons are statistically significant. It also does not discriminate between situations where the observations are all consistent i.e. statistical significance provides support for the validity of the instrument, versus those where one or more observations appear to be inconsistent i.e. statistical significance may or may not provide support for the validity of the instrument. Given the multiple issues identified regarding tests of statistical significance in this context, we recommend that caution should be exercised when interpreting any measures of a psychometric property which rely on tests of statistical significance.

The EuroQol Group have approved the development of “bolt-ons/dimension extensions” [24]. These instruments will permit the addition of extra dimensions to the standard EQ-5D instrument in order to directly capture other issues of importance to patients. How precisely these bolt-ons are approached remains to be seen, but this may be a route to addressing symptoms such as incontinence which are not captured directly by any of the current dimensions. This review has not identified any strong evidence to suggest that the impact of incontinence is not adequately captured indirectly through the existing dimensions, although it did not examine content validity directly. A review by Lin *et al* identified several candidate areas for bolt-ons by comparing the content of disease specific preference based measures to that of the EQ-5D across a wide variety of disease areas [25]. Despite including one paper in patients with urinary incontinence and another in patients with overactive bladder, incontinence was not identified by Lin *et al.* as a potential candidate for bolt-ons to the EQ-5D. One of the key advantages of the EQ-5D, which may be threatened by the addition of bolt-on dimensions, is that it provides a generic measure of HRQoL that allows decision makers to apply a consistent approach to economic evaluation across multiple disease areas.

## Conclusions

This review provides a narrative summary of the evidence available on the appropriateness of the EQ-5D instrument in assessing the health impact of UI. The EQ-5D was generally found to perform well on tests of construct validity, responsiveness and reliability, although no definitive conclusion can be made on its appropriateness based on these measures alone.

## Abbreviations

AQoL: Assessment of quality of life; BFLUTS: Bristol female lower urinary tract symptoms questionnaire; DIS: Detrusor instability scores; EQ-VAS: Visual analogue scale which accompanies the EQ-5D descriptive system; GP: General practice; HRQoL: Health related quality of life; HUI3: Health utilities index mark 3; ICSQol: International continence society – Benign prostatic hyperplasia study quality of life instrument; IIQ-7: Incontinence impact questionnaire-short form; I-PSS: International prostate symptom score; I-QOL: Incontinence specific quality of life questionnaire; KHQ: King’s health questionnaire; NASHA/Dx: Non-animal-stabilized hyaluronic acid/dextranome; PGI: Patient generated index; QALY: Quality adjusted life year; RCT: Randomised controlled trial; SF-36: Medical outcomes study 36-item short-form health survey; SF-6D: Classification for describing health derived from a selection of SF-36 items; SSI: Symptom severity index; S/UIQ: Stress and urge incontinence questionnaire; TTO: Time trade off; TVT: Tension-free vaginal tape; TVT-O: Tension-free vaginal tape obturator; UDI-6: Urogenital distress inventory-short form; UI: Urinary incontinence; UISS: Urinary incontinence severity score; UK: United Kingdom; VAS: Visual analogue scale; 15-D: Fifteen dimension generic instrument.

## Competing interests

The authors declare that they have no competing interests.

## Authors’ contribution

SD and AW contributed to the overall design of the review, interpretation of the results and drafting of the manuscript. SD was also responsible for identifying included studies and extracting and summarising study data. Both authors read and approved the final manuscript.

## Authors’ information

SD is a Senior Lecturer in Health Economics and Deputy Director of the NICE Decision Support Unit. AW is a Professor in Health Economics and Director of the NICE Decision Support Unit.

## Supplementary Material

Additional file 1 **Medline search strategy.** Details of the search strategy for the MEDLINE database. Click here for file
