# Supplementary material for: A review of the psychometric performance of the EQ-5D in people with urinary incontinence
Source: Health Qual Life Outcomes. 2013 Feb 18;11:20. doi: 10.1186/1477-7525-11-20 (PMC3622573; doi:10.1186/1477-7525-11-20)
Supplement: Additional file 1 — Medline search strategy. Details of the search strategy for the MEDLINE database. [file 1477-7525-11-20-S1.pdf]

MEDLINE search strategy:

Database: Ovid MEDLINE(R) In-Process & Other Non-Indexed Citations and Ovid MEDLINE(R) <1950 to Present>

Search Strategy:

----

1 exp Urinary Incontinence/ (22268)

2 bladder weakness.mp. (4)

3 bladder control.mp. or Urinary Bladder, Overactive/ (1410)

4 1 or 2 or 3 (23286)

5 (euroqol or euro qol or eq5d or eq 5d or eq-5d or (euro adj qol) or (eur adj qual) or (eq adj 5d)).mp. [mp=title, original title, abstract, name of substance word, subject heading word, unique identifier] (1928)

6 4 and 5 (20)
